# Evolution of cis- and trans-regulatory divergence in the chicken genome between two contrasting breeds analyzed using three tissue types at one-day-old

**DOI:** 10.1186/s12864-019-6342-5

**Published:** 2019-12-05

**Authors:** Qiong Wang, Yaxiong Jia, Yuan Wang, Zhihua Jiang, Xiang Zhou, Zebin Zhang, Changsheng Nie, Junying Li, Ning Yang, Lujiang Qu

**Affiliations:** 1 State Key Laboratory of Animal Nutrition, Department of Animal Genetics and Breeding, National Engineering Laboratory for Animal Breeding, College of Animal Science and Technology, China Agricultural University, Beijing, China; 2 Key Laboratory for Sustainable Utilization of Marine Fisheries Resources, Ministry of Agriculture and Rural, Yellow Sea Fisheries Research Institute, Chinese Academy of Fishery Sciences, Qingdao, China; 3 Institute of Animal Science, Chinese Academy of Agricultural Sciences, Beijing, China; 4 Department of Animal Science and Technology, Qingdao Agricultural University, Qingdao, China; 5 Department of Animal Sciences, Center for Reproductive Biology, Veterinary and Biomedical Research Building, Washington State University, Pullman, USA; 6 College of Animal Sciences and Veterinary Medicine, Huazhong Agricultural University, Wuhan, China

**Keywords:** Cis, Trans, Regulation, RNA-seq, Allele-specific expression

## Abstract

**Background:**

Gene expression variation is a key underlying factor influencing phenotypic variation, and can occur via cis- or trans-regulation. To understand the role of cis- and trans-regulatory variation on population divergence in chicken, we developed reciprocal crosses of two chicken breeds, White Leghorn and Cornish Game, which exhibit major differences in body size and reproductive traits, and used them to determine the degree of cis versus trans variation in the brain, liver, and muscle tissue of male and female 1-day-old specimens.

**Results:**

We provided an overview of how transcriptomes are regulated in hybrid progenies of two contrasting breeds based on allele specific expression analysis. Compared with cis-regulatory divergence, trans-acting genes were more extensive in the chicken genome. In addition, considerable compensatory cis- and trans-regulatory changes exist in the chicken genome. Most importantly, stronger purifying selection was observed on genes regulated by trans-variations than in genes regulated by the cis elements.

**Conclusions:**

We present a pipeline to explore allele-specific expression in hybrid progenies of inbred lines without a specific reference genome. Our research is the first study to describe the regulatory divergence between two contrasting breeds. The results suggest that artificial selection associated with domestication in chicken could have acted more on trans-regulatory divergence than on cis-regulatory divergence.

## Background

Numerous transcriptional regulatory factors, which can be classified into cis-regulatory elements and trans-regulatory factors, regulate gene expression [1]. Cis-regulatory elements, such as promoters, enhancers, and silencers, are regions of non-coding DNA, which regulate the transcription of nearby genes. In contrast, trans-regulatory factors regulate (or modify) the expression of distant genes by combining with their target sequences [1, 2]. In most cases, complex interactions between cis-regulatory sequences and trans-acting factors control gene expression [3, 4].

Cis- and trans-regulatory elements are thought to vary based on key genetic and evolutionary properties [5, 6]. In diploid individuals, cis-regulatory elements regulate gene expression in an allele-specific manner. Cis-regulatory variation heterozygotes express allelic imbalances at the transcriptional and translational levels. By comparison, trans-regulatory factors interact with target sequences to regulate both alleles [1]. Trans-regulatory divergence is enriched for dominant effect, while the effects of cis-regulatory variants are additivity [6, 7]. Beneficial cis-regulatory variants are more likely to be enriched to fixation in the course of evolution, because the additive effects expose rare alleles to selection [5].

Both cis- and trans-regulatory variation are play key roles in phenotypic variation [1, 8–10]. Previous work in a wide range of species, including *Drosophila* [7], mouse [11, 12] and *Coffea* [13], have used allele-specific expression (ASE) analysis [14] to distinguish between cis- and trans-regulatory divergence (Table 1). However, gene regulatory divergence in birds could be different from gene regulatory divergence in mammals, insects, or plants, considering some genetic mechanisms involved in ASE in birds are unique. For instance, genomic imprinting has been observed in mammals and some plants [15–17], but seems largely absent in birds assessed to date [18–20]. Dosage compensation exists in some diploid species to buffer the effect of copy number difference of genes on the sex chromosome [21–23], but it has been reported to be incomplete in birds [24–28]. Therefore, it is critical to investigate gene regulatory divergence in birds.

**Table 1:** Studies that have classified gene regulatory divergence in genomes

Species	Tissue	Sex	Cis	Trans	Cis and trans	Conserved and ambiguous	Method	Citation
Drosophila	Whole fly	Female	12.4%	30%	35%	22.6%	Hierarchical statistical analyses	McManus et al., 2010
Mouse	Liver	Male	14%	0.6%	17.4%	68%	Maximum likelihood based approach	Goncalves et al., 2012
Mouse	Testis	Male	24%	9%	44%	23%	Hierarchical statistical analyses	Crowley et al., 2015
Coffea^a^	Leaf		15.5%	18.5%	17.5%	48.0%	Hierarchical statistical analyses	Combes et al., 2015
			14.5%	18.3%	16.6%	50.6%		
Chicken^b^	Brain	Female	3.45%	3.70%	4.88%	87.99%	Hierarchical statistical analyses	This article
		Male	3.75%	4.86%	4.37%	87.01%		
	Liver	Female	7.41%	12.92%	16.15%	63.53%		
		Male	8.31%	13.93%	17.07%	60.70%		
	Muscle	Female	5.60%	15.80%	10.79%	67.82%		
		Male	4.72%	16.73%	11.58%	66.99%		

^a^This article contains two crosses (cross C × E and cross E × C)

^b^This study contains two crosses (cross 2 and cross 3), and we took the cross 2 as an example

Chicken is a model animal for studies on birds, and a remarkable example of rapid phenotypic divergence, with artificial selection resulting in major size, behavioral, and reproductive differences among breeds [29]. Previous studies have identified frequent ASE among different chicken breeds [19, 20]. The rapid change under domestication offers a unique model for revealing the relative importance of the cis- and trans-regulatory variation underlying phenotypic change. We used reciprocal crosses of White Leghorn (WL), a key layer breed selected for its high egg output, and Cornish Game breeds (CG), a cornerstone broiler breed selected for its rapid growth and muscle development [30], to assess the role of different forms of regulatory variation in the brain, liver, and muscle tissue of 1-day-old males and females.

## Results

### The profile of the parental genomes and gene expression in different tissues, sexes of progenies

**Fig. 1:**
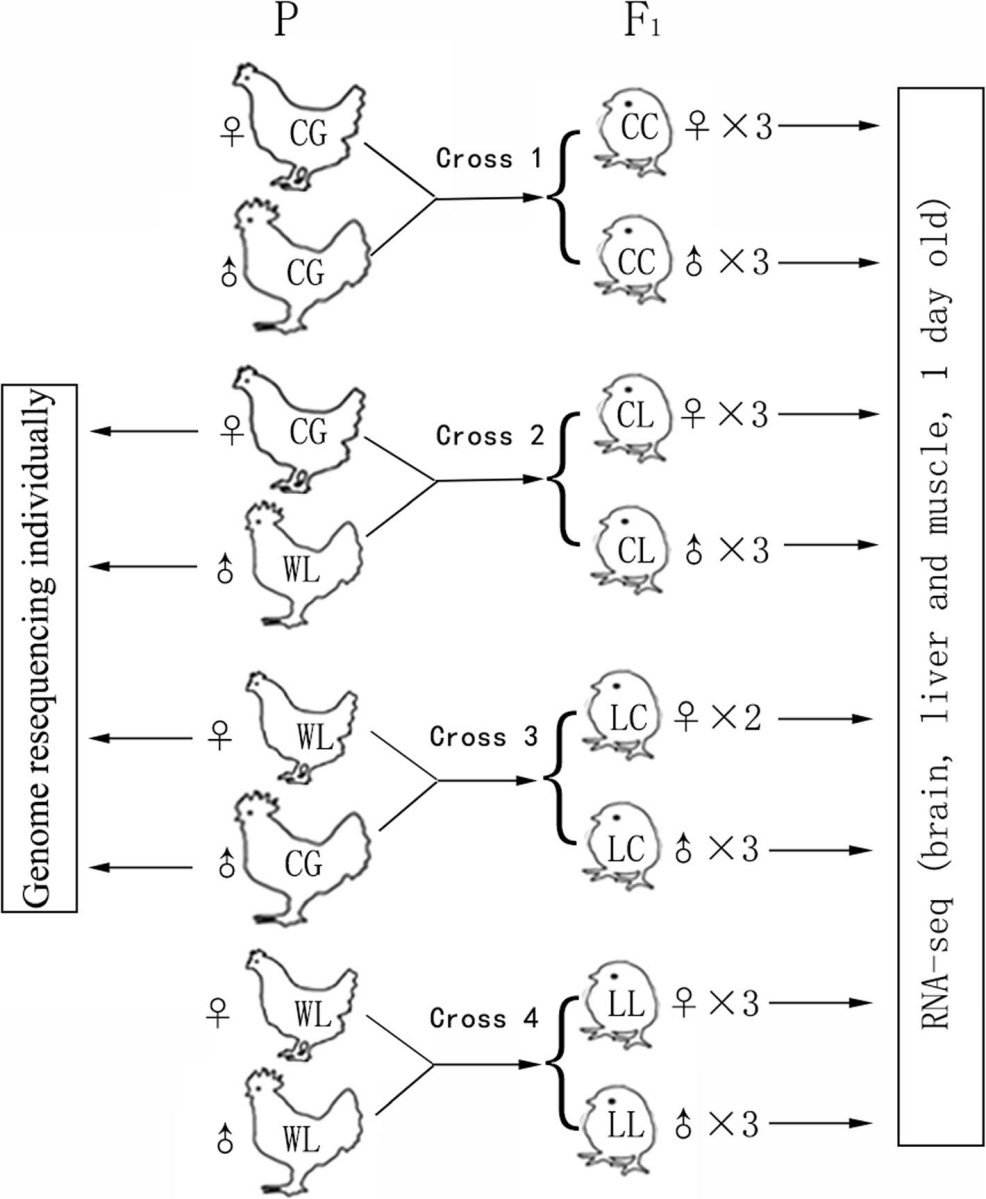
Cross design. Cornish-Game (CG) and White-Leghorns (WL) were used to generate purebreed and hybrid progenies. There were four crosses, Cross 1: CG × CG, cross 2: CG × WL, cross 3: WL × CG, and cross 4: WL × WL (the female parent is listed first)

The two inbred chicken strains, CG and WL, which exhibit major differences in growth rate, egg production, and behavior, were used to generate purebreed and reciprocal hybrid F1 progenies (Fig. 1). To identify breed-specific variants, we sequenced the genes of four parents of the two reciprocal crosses, recovering on average 100.73 million pair-end reads per sample after quality control. We identified on average 4.74 million single-nucleotide polymorphisms (SNPs) per parental genome, which were used to generate simulated parental genomes. We picked SNPs that were homozygous in each parental bird but different from each other in the same cross (heterozygous in the hybrid progenies), resulting in two heterozygous SNP lists with 1.4 million heterozygous SNPs on average for the two reciprocal crosses, individually, to identify the allele-specific RNA-Seq reads of the offspring in the following steps.

**Fig. 2:**
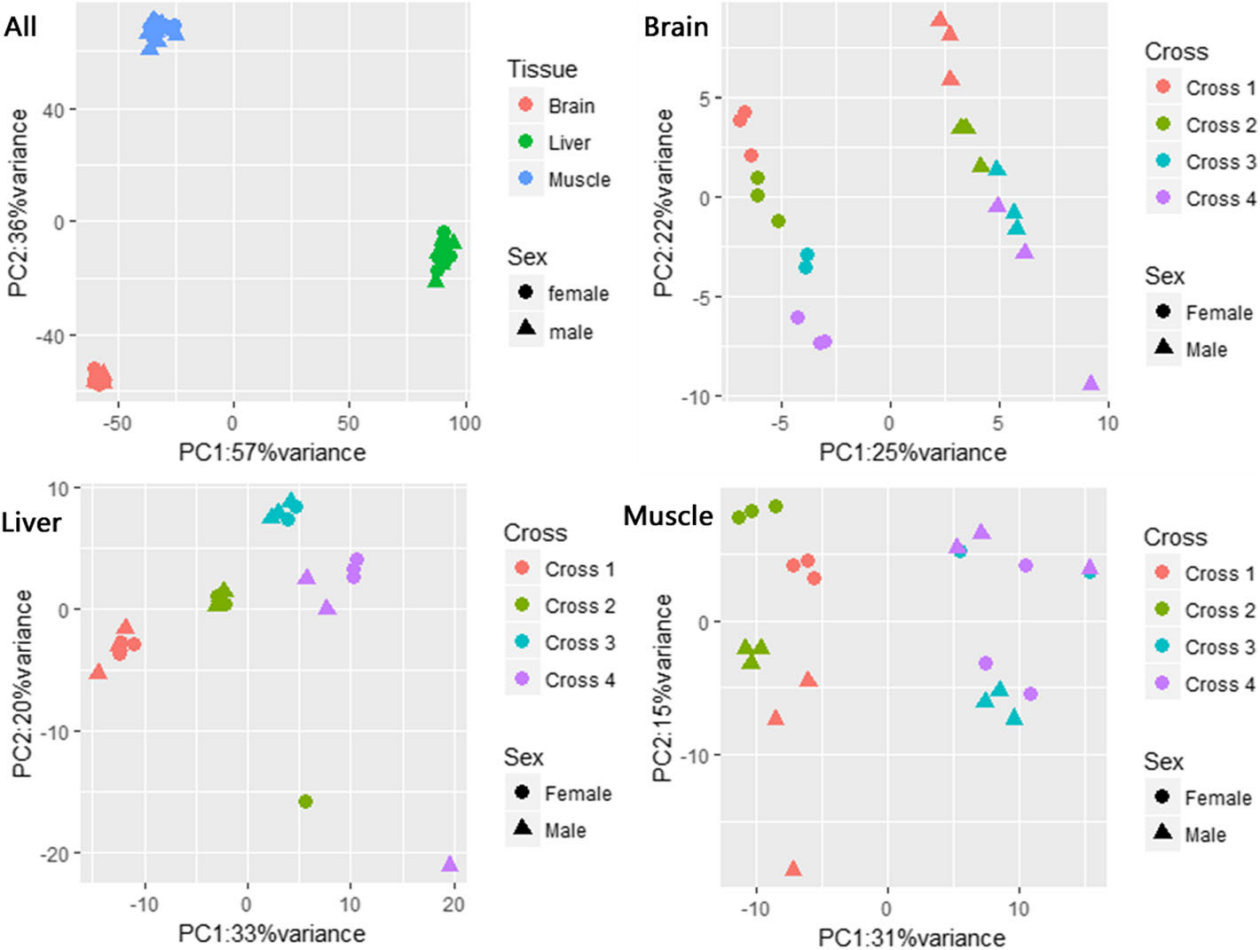
Principal Component Analysis of RNA-Seq data. Each point represents one sample, with shape indicating sex, color indicating tissue (All) or cross (Brain, Liver, and Muscle). In this step, information on genes on the Z chromosome has been excluded

For each hybrid cross, we collected RNA-Seq data from the brain, liver, and muscle tissue of three male and three female F1 progenies 1 day post-hatching. On average, we recovered 29.17 million mappable reads per sample. To eliminate the effect of the sex chromosomes, we removed all Z and W genes from our analysis and focused entirely on autosomal loci. We observed significant differences in gene expression among different tissues, between sexes, and between parents-of-origin (Fig. 2). Tissue was the most significant factor influencing gene expression, sex played a leading role in the brain, strain influenced gene expression of liver the most, while in the muscle, the parent-of-origin seemed the most powerful because samples were divided into two parts based on mother origin. Consequently, we retained all three variables in our subsequent analyses, resulting in 12 treatment groups, comprised of three tissues, two sexes, and two reciprocal crosses in the present study.

### An effective pipeline was applied for the allele-specific expression analysis

To identify the parental origin of the mRNA of the offspring, we explored a novel pipeline using the ‘asSeq’ package in R [31]. Briefly, a set of R scripts was available for genotype phasing based on the 1.4 million heterozygous SNPs identified in the preceding step. Approximately 2% of the SNPs mentioned above were located in the exon region. The high number of SNPs increased the chances that an RNA-seq read could overlap with a heterozygous genetic marker to enable its identification as an allele-specific read.

To validate the accuracy of our ASE pipeline, we generated two artificial hybrid F1 libraries. Specifically, we concatenated two male brain RNA-Seq fastq files from cross 1 and cross 4, which had roughly equal read depths. We also concatenated two female liver samples in the same manner. The two simulated hybrid libraries and four original purebred libraries were handled similar to the other hybrid libraries, using the heterozygous SNP lists of both cross 2 and cross 3. We compared the expression ratio of two simulated alleles (CG/WL) to the real expression ratio of two samples (CG/WL) for each gene. A strong correlation between the two measurements was observed (Additional file 1: Figure S1), indicating that our ASE analysis pipeline was robust. Since our pipeline only counted the local reads containing the heterozygous SNPs, we further assessed the expression fold change (CG/WL) correlation between the local reads method and the method of counting total reads using edgeR [32–34]. The correlation was also strong (Additional file 1: Figure S2). These results demonstrated the feasibility of our pipeline.

### Genes were classified into different categories based on the type of regulatory divergence

A total of 24,881 genes from Ensembl v87 annotation were analyzed. Approximately a fifth of the genes contained heterozygous SNPs and were expressed in our progeny samples (Additional file 1: Table S1). For the genes containing heterozygous SNPs, we observed significant expression differences (*p*-value < 0.05, binomial test corrected for multiple comparisons by q-value method) between the purebred females (cross 1 vs. cross 4), in 14.71% in the brain, 36.45% in the liver, and 38.38% in muscle (consider the heterozygous SNP list of cross 2, for example). In males, 17.64% of the genes in the brain, 41.87% of the genes in the liver, and 37.84% of the genes in muscle were expressed significantly differentially (Additional file 1: Table S1).

**Fig. 3:**
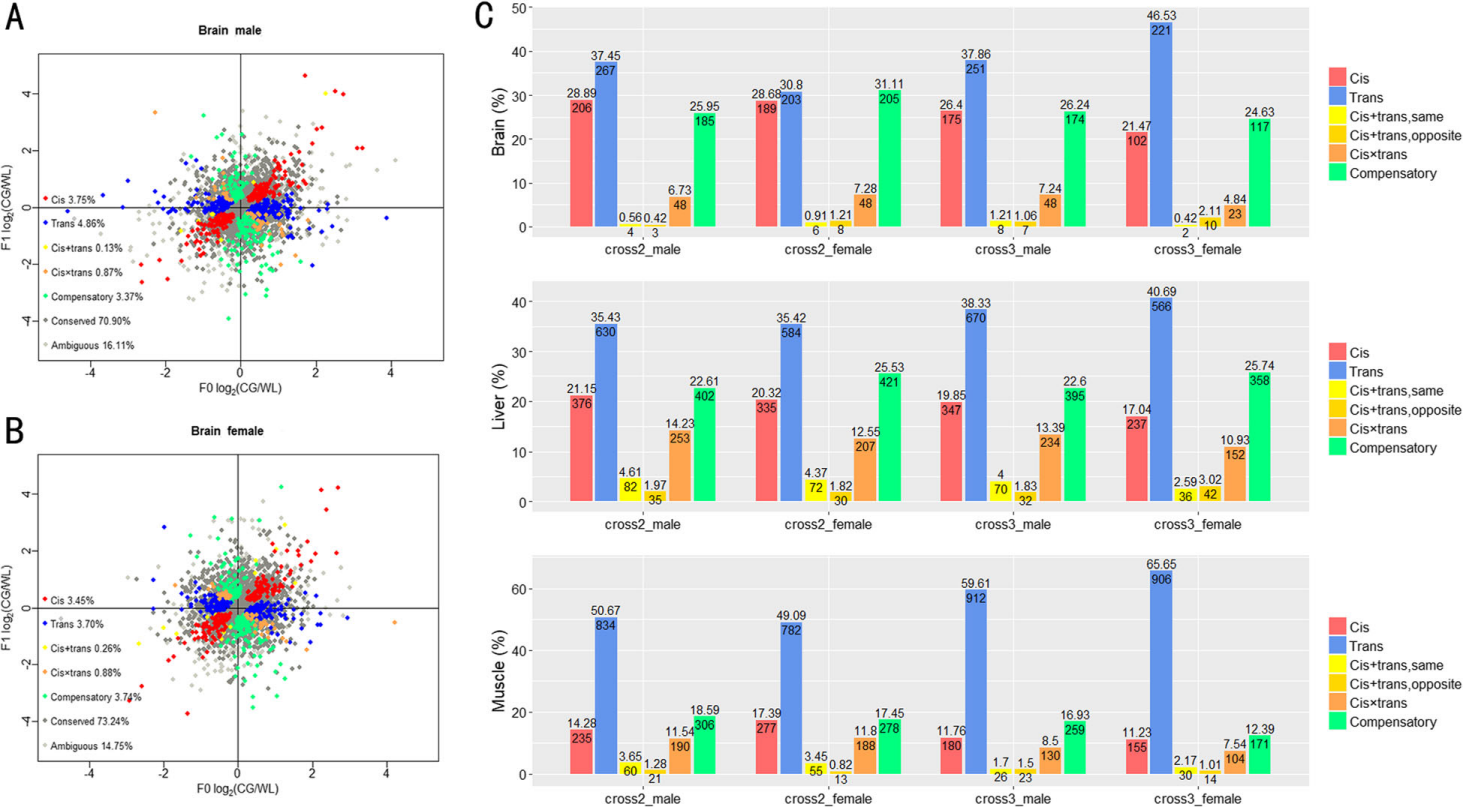
Classification of genes according to the expression pattern of purebreed and hybrid data sets. Consider the male brain **a** and the female brain **b** of cross 2, for example (for the other groups, see Additional file). Each point represents a single gene and is color-coded according to its regulatory category. The coordinate position shows the average log2 expression fold-change between the alleles in the hybrids (y-axis) and between the two purebreeds (x-axis). The proportion of each category is summarized in the bar graph **c**, where we removed the conserved and ambiguous genes, and further subdivided the cis + trans category genes into two categories, based on whether the cis and trans variants acted in the same direction or in opposite directions. The number above the bar represents the proportion of genes in the regulatory category, and the number on the bar represents the gene count of the category

Expressed genes were classified into different categories based on the type of gene regulatory divergence [7, 35, 36] (Fig. 3a, b, Table 1, Additional file 1: Figure S3-S5). Most genes exhibited conserved or ambiguous expression, as expected, considering the relatively recent divergence time of the two breeds investigated. More than 70, 40%, and approximately 50% of the genes in the brain, liver, and muscle, respectively, were classified as conserved. Nonetheless, we observed substantial cis- and trans-variation in the hybrid crosses. There was a higher proportion of trans-regulated gene expression variations than cis-regulated gene expression in most tissues and across both sexes, particularly in muscle (Fig. 3c).

Genes regulated by both cis- and trans-regulatory variations were divided into four categories, including “cis + trans (same)”, “cis + trans (opposite)”, “cis × trans”, and “compensatory”. Genes classified as “cis + trans (same)” show cis and trans-variations acting in a similar direction, while genes classified into the other three categories show cis and trans-variations acting in opposite directions, with different expression trends on the two alleles. We observed the latter pattern more frequently, and most genes were classified as “compensatory” (Fig. 3c).

The gene proportions in each regulatory category were similar among different tissues and between different sexes, except for some variation between the muscle and the other two tissues (Fisher’s exact test, Additional file 1: Table S2). Unexpectedly, we observed only few loci with consistent cis- or trans-regulatory divergence across different groups (Additional file 1: Figure S6). The stable cis- or trans-regulatory divergence genes seem to play key roles in phenotypic divergence. For example, *IGFBP2*, *TGFBI*, *PDGFRL*, and *IGF2R* all showed significant expression bias between the two breeds investigated. The genes are associated with chicken growth, which could explain the difference in growth rate between the two breeds (Additional file 1: Table S3).

### Genes regulated by trans-acting variation exhibit greater sequence conservation

**Fig. 4:**
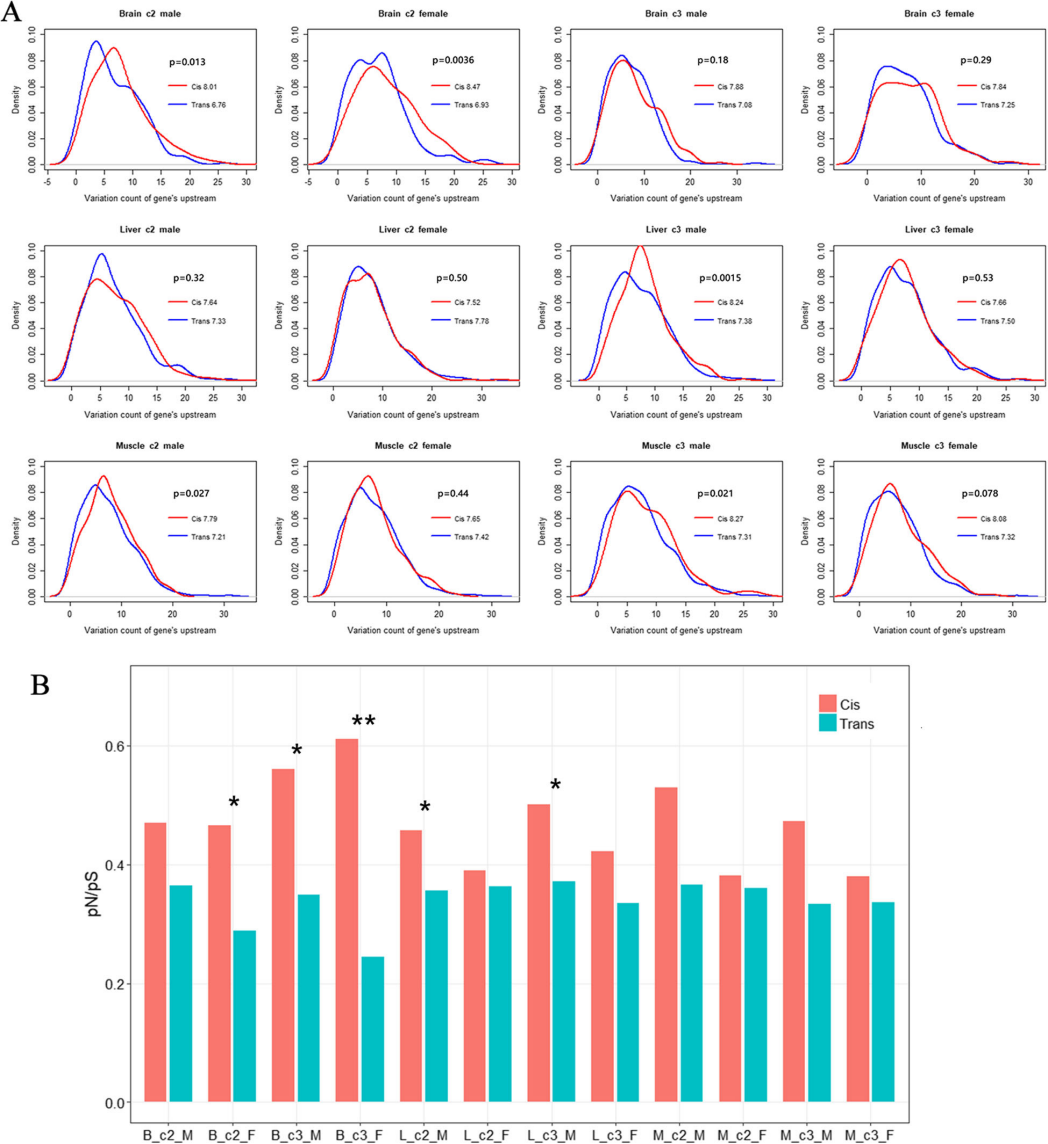
Sequence conservation analysis of the cis- and trans-regulatory divergence genes. **a** The probability density (y-axis) of variation count (x-axis) 1-kb of DNA upstream of each gene’s transcription start site. The number following the regulatory category name in the legend refers to the mean value of variation count of all genes in this category. The p-value above the legend was obtained using the Mann-Whitney U test. **b** The pN/pS values in cis- and trans-regulatory divergence genes. The y-axis refers to the mean value of all genes in the category. Significance of the difference between the two regulatory categories is labeled above the bar (* *p* < 0.05, t-test; ** *p* < 0.01, t-test)

We counted the number of variants located 1 kb upstream of transcription start sites of each gene using the genome data of the four parents. The results showed greater variations upstream of cis-regulatory divergence genes than upstream of trans-acted genes in all samples (Fig. 4a).

The ratio of the number of non-synonymous SNPs to the number of synonymous SNPs (pN/pS) in the coding sequences of each gene was calculated in the present study. The pN/pS values in genes regulated by trans-variants were lower than the pN/pS values of genes regulated by cis-variants in all samples (Fig. 4b, Additional file 1: Figure S7–S8).

## Discussion

Previous studies on regulatory divergence genes did not select identical time points from the embryo to adult stages [7, 11, 12]. Genes are expressed differentially across different developmental stages [37]; therefore, different results would be obtained from the regulatory divergence genes across different development stages. We selected 1-day-old chicken because it is a critical stage in their development when they transition from embryo to chicks, and genes responsible for growth and immunity begin to be expressed [38, 39].

Considering the relatively short divergence time, the two inbred chicken strains are not similar to mouse inbred lines, which exhibit high levels of consistency within genomes. To enhance the reliability of our results, we have improved our analysis pipeline. First, the SNP list we used to identify the parental origin was filtered strictly from the re-sequencing data of the four parents. The SNPs were statistically homozygous in each parent; and therefore, heterozygous in each hybrid offspring. Secondly, we counted the total number of reads covering at least one SNP marker across the whole transcript instead of counting the read number of each SNP. Compared with the method using the existing strain-specific reference genomes, our pipeline could improve the accuracy of parental origin identification for heterozygous SNPs in hybrid offspring because we sequenced their parents directly. The SNPs were used to mark the parental origins of the alleles of each gene, which increased the accuracy of classification. However, it also resulted in a limited number of genes that could be studied. Nevertheless, our study offers an example for addressing similar situations where there is no specific reference genome for different strains.

Although chicken domestication occurred several thousand years ago, commercial populations were established only over the last 200 years [29]. In our study, most genes exhibited conserved or ambiguous expression, and more trans-regulatory variants compared to cis-regulatory variants, which could be attributed to the relatively short differentiation time between WL and CG. In theory, the pleiotropic effects of trans-regulatory mutations would result in selection to eliminate the most deleterious trans-acting mutations [40]. In contrast, we could expect a large proportion of cis-regulatory mutations to be largely neutral, and therefore, to accumulate over time [9, 41]. The large proportion of trans-regulatory mutations observed in the present study suggest that artificial selection has primarily acted on trans-regulatory mutations, but the neutral cis-regulatory mutations have not accumulated substantially over the relatively short period since the breeds were established.

Genes regulated by both cis- and trans-variations act in opposite directions more often than not, and most genes were classified as “compensatory” in the present study. This finding is consistent with the results of a previous study on house mice [36], in which the cis- and trans-variants tended to act convergently to maintain the stability of gene expression [11, 42]. Despite the lack of a complete dosage compensation mechanism on the sex chromosome [24–28], an extensive compensatory trend persists in the chicken genome.

There were few loci with consistent cis- or trans-regulatory variation among different tissues and between different sexes. The result is consistent with the findings of some previous ASE analyses, which suggested that rare ASE genes are expressed consistently across tissues [43, 44]. However, the cis- and trans-regulatory divergence classification is much more complex than the ASE analysis. Gene expression is characterized by spatiotemporal specificity. It is always controlled by the interaction of cis-regulatory DNA sequences and trans-regulatory factors, which could complicate the identification of regulatory divergence. Statistical methods would not accurately classify them based on limited expression information. However, statistical result would still be reliable and valuable for subsequent analyses.

Cis-regulatory elements are primarily located upstream of coding sequences. Our results are consistent with the findings of a recent study in *Drosophila* [7], which detected greater variants 1 kb upstream of transcription start sites of cis-regulatory divergence genes than upstream of transcription start sites trans-acted genes, suggesting that our classification results were reliable. In addition, genes regulated by trans-variants showed a lower pN/pS value than cis-acting genes. The pN/pS value has been used to assess the degree of selective constraint. Genes under high selective constraint are expected to have lower pN/pS values [45, 46]. Our results suggest that trans-regulatory divergence genes were subjected to high selective constraint in the course of chicken domestication and could have been under stronger artificial selection, which is consistent with the findings of similar studies in mice [11] that reported that trans-regulated genes exhibited greater sequence conservation based on the computed Genomic Evolutionary Profiling scores for each exon.

## Conclusions

In the present study, we present a pipeline for exploring ASE in the hybrid progenies of inbred lines without a specific reference genome. Using the genome sequences of parents and RNA-seq data of offspring, we classified the genes expressed in the chicken genome into different categories based on the type of regulatory divergence involved. More instances of trans-regulatory divergence than instances of cis-regulatory divergence were observed due to the relatively short history of divergence in the two parental breeds. Considerable compensatory cis- and trans-regulatory changes exist in the chicken genome. The sequence conservation analysis results suggested that artificial selection associated with domestication could have potentially acted on genes regulated by trans-variations in the course of the establishment of commercial chicken breeds.

## Methods

### Samples

The inbred chickens used in our study were obtained from the National Engineering Laboratory for Animal Breeding of the China Agricultural University. We collected brachial vein blood from 4 parents of two reciprocal crosses and extracted DNA using the phenol-chloroform method according to standard protocols. Three tissues, including brain tissue, liver tissue, and breast muscle tissue were collected from 23 1-day-old chickens. All the tools and equipment used for sampling were sterilized by heat or ultraviolet rays.

Our animal experiments were approved by the Animal Care and Use Committee of China Agricultural University. All the animals were fed and handled according to the regulations and guidelines established by this committee, and all efforts were made to minimize suffering. The 4 parental chickens of the two reciprocal crosses were released after collected brachial vein blood, and the 23 1-day-old chickens were beheaded before we collected tissues.

The tissues were deposited in RNAlater (Invitrogen, Carlsbad, CA, USA), an RNA stabilization solution, at 4 degrees Celsius for one night and then moved to − 20 degrees Celsius refrigerator. Total RNA was extracted from the tissue samples using Trizol reagent (Invitrogen, Carlsbad, CA, USA) according to manufacturer’s instructions. The DNA and RNA quality was assessed using a NanoDrop 2000 spectrophotometer (Thermo Fisher Scientific Inc., USA) and agarose gel electrophoresis.

### DNA & RNA sequencing and data alignment

Whole-genome sequencing of parent genomes and RNA-seq of offspring were performed on the Illumina HiSeq 2500 platform (Illumina Inc., San Diego, CA, USA). Library construction and sequencing were performed according to manufacturers’ instructions (TruSeq DNA Sample Prep Kit, TruSeq RNA Sample Prep Kit, TruSeq PE Cluster Kit v3-cBot, and TruSeq SBS Kit v3, Illumina). Both DNA and RNA were sequenced with paired-end 100-bp reads with a 300-bp insert. All sequencing data were filtered using an NGS QC Toolkit v2.3 [47] according to default parameters.

To ensure the accuracy of RNA-seq data alignment, we simulated four parental genomes. The re-sequencing data of the four parents were mapped to the chicken reference genome (Gallus_gallus-5.0, http://hgdownload.soe.ucsc.edu/downloads.html#chicken) using the Burrows-Wheeler Aligner v0.7.15 [48]. The BAM files were sorted and duplicate reads removed using Picard toolkit (https://github.com/broadinstitute/picard). The Genome Analysis Toolkit v3.6 [49] was used for SNP calling. Nucleotides from the reference genome were substituted if the mutant base was supported by more reads than the original reference base, which was performed using VCFtools v0.1.13 [50]. The four simulated parental genomes were used to replace the reference genome in the RNA-Seq data alignment of the hybrid crosses. For each hybrid cross, we identified SNPs between two parents that were homozygous in each parent with > 10 supporting reads from the re-sequencing data. The SNP list divided each hybrid offspring genome into two parts based on the parent-of-origin.

The RNA-Seq data alignment was performed using STAR v2.5.3a [51]. Based on the SNP list between every two parents, we counted the allele-specific reads from the two parts of each hybrid offspring at exon set level, using the ‘asSeq’ package in R [31]. Specifically, we counted the total number of reads covering at least one SNP across the whole exon set. In the case of one read containing more than one SNPs, we set the parameter of prop.cut to 0.9, that is, we assigned a read to one of the two parental alleles if the proportion of those heterozygous SNPs suggested the read that was from that allele was greater than 0.9. In practice, this ensures all the SNPs on one read are consistent. If not, they would be discarded. We then collapsed counts at the exon level to the gene level according to the Ensembl gene annotation file (ftp://ftp.ensembl.org/pub/release-91/gtf/gallus_gallus). We filtered the expressed genes using the following criterion: for each sex and each tissue, the total reads of the three purebred offspring and the three hybrid offspring have to be between 6 and 1000. The read counts of each sample were further normalized based on the sum of reads that could be mapped to the whole genome.

One male muscle sample of cross 3 was removed because its expression pattern was abnormal. We speculated that it could have been mixed with another cross by error.

### Classification of different regulatory categories

To categorize regulatory variations, we referenced the methods applied in the study of regulatory divergence in *Drosophila* [7] and house mouse [36]. The binomial test was used to identify differential expression both between the two purebred progenies (P) and between the two alleles of the hybrid progenies (H). Fisher’s exact test was used to evaluate the breed-specific RNA abundance ratio differences between the P and H data sets to detect any trans effects (T). False discovery rate was controlled by adopting a method of q-value estimation [52] to correct the *p*-values of both the binomial test and the Fisher’s exact test. A difference was considered significantly different when q < 0.05. The expressed genes were classified into eight categories according to the following criteria:

1. Cis: Significant difference in P and H, no significant difference in T.
2. Trans: Significant difference in P, but not H, significant difference in T.
3. Cis + trans (same): significant difference in P, H. and T, the log2-transformed strain-specific ratios in P and H have the same sign, and the difference in P is higher than the difference in H.
4. Cis + trans (opposite): significant difference in P, H and T, the log2-transformed strain-specific ratios in P and H have the same sign, and the difference in H is higher than the difference in P.
5. Cis × trans: significant difference in P, H and T, and the log2-transformed strain-specific ratios in P and H have the opposite sign.
6. Compensatory: Significant difference in H, but not in P, and significant difference in T.
7. Conserved: No significant difference in H, P, and T.
8. Ambiguous: All other patterns.

### Sequence conservation analysis

Re-sequencing data from four parents were used to study the sequence conservation of cis- and trans-regulatory divergence genes. The pN/pS ratio of the coding sequence and the number of variants in 1 kb upstream from the transcription start site were calculated using the results of SNP annotation performed using SnpEff [53]. Non-synonymous mutation contains a missense variant, start codon lost, start codon gained, stop codon lost, and stop codon gained. Synonymous mutation refers to the variant in the coding region causing a codon that produces the same amino acid.

## Supplementary information

**Additional file 1:** **Figure S1.** Assessment of our analysis pipeline for estimating allele specific expression. **Figure S2.** Assessment of our local reads method for estimating allele specific expression. **Figure S3.** Classification of genes in brain. **Figure S4.** Classification of genes in liver. **Figure S5.** Classification of genes in muscle. **Figure S6**. Intersection of different groups of cis- and trans- regulatory genes. **Figure S7.** The ratio of the numbers of non-synonymous SNPs to the numbers of synonymous SNPs (pN/pS) in different groups of cross 2. **Figure S8.** The ratio of the numbers of non-synonymous SNPs to the numbers of synonymous SNPs (pN/pS) in different groups of cross 3. **Table S1.** The summary of differential expression genes in hybrid and purebred progenies **Table S2.** The difference of gene proportion of each categories between different groups **Table S3.** The gene list of intersection of each group

## Data Availability

The datasets generated and/or analyzed during the current study are available in the NCBI BioProject (https://submit.ncbi.nlm.nih.gov/subs/bioproject/) with accession number PRJNA591354.

## Abbreviations

ASE: Allele specific expression
CG: Cornish Game
WL: White Leghorn
