# Supplementary material for: Evolution of cis- and trans-regulatory divergence in the chicken genome between two contrasting breeds analyzed using three tissue types at one-day-old
Source: BMC Genomics. 2019 Dec 5;20:933. doi: 10.1186/s12864-019-6342-5 (PMC6896592; doi:10.1186/s12864-019-6342-5)
Supplement: Supplementary file 1 — Additional file 1: Figure S1. Assessment of our analysis pipeline for estimating allele specific expression. Figure S2. Assessment of our local reads method for estimating allele specific expression. Figure S3. Classification of genes in brain. Figure S4. Classification of genes in liver. Figure S5. Classification of genes in muscle. Figure S6. Intersection of different groups of cis- and trans- regulatory genes. Figure S7. The ratio of the numbers of non-synonymous SNPs to the numbers of synonymous SNPs (pN/pS) in different groups of cross 2. Figure S8. The ratio of the numbers of non-synonymous SNPs to the numbers of synonymous SNPs (pN/pS) in different groups of cross 3. Table S1. The summary of differential expression genes in hybrid and purebred progenies Table S2. The difference of gene proportion of each categories between different groups Table S3. The gene list of intersection of each group [file 12864_2019_6342_MOESM1_ESM.docx]

**Supplemental figures**


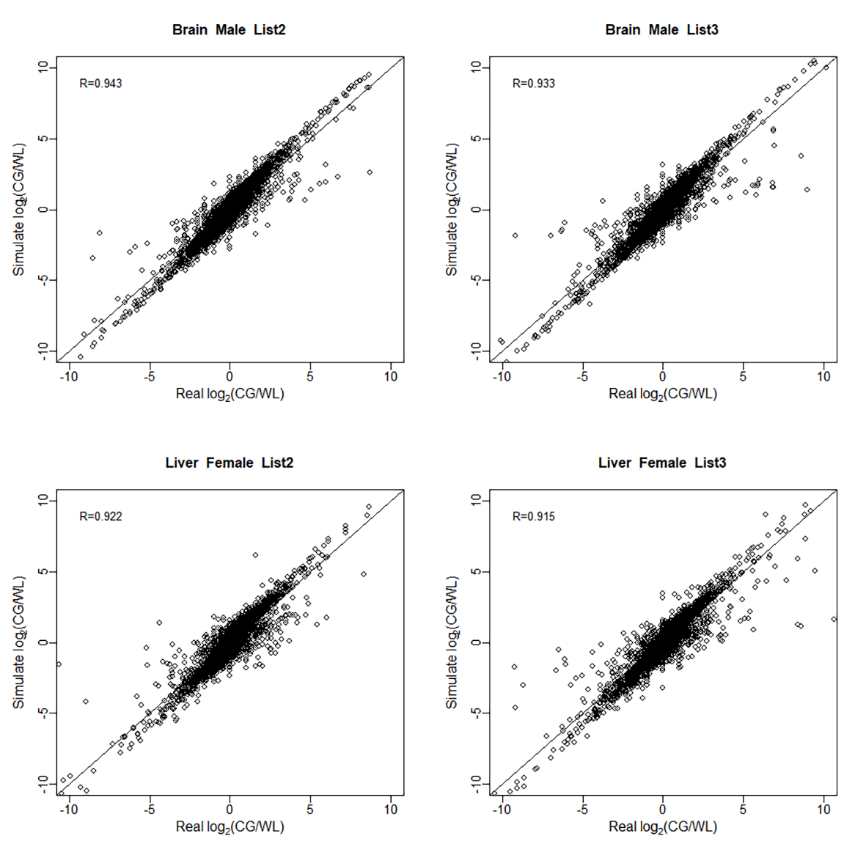


**Figure S1 Assessment of our analysis pipeline for estimating allele specific expression.** The two plots above were male brain samples, and the two plots below were female liver samples. Reads samples in the two left plots were counted using the SNP list of cross 2, and samples of the two right plots using the SNP list of cross 3. Each point represents one gene. The x-axis shows fold change of two purebred individuals, and the y-axis shows fold change of the simulated F1 alleles. Pearson correlation coefficient was calculated and tagged in the top left of each plot.


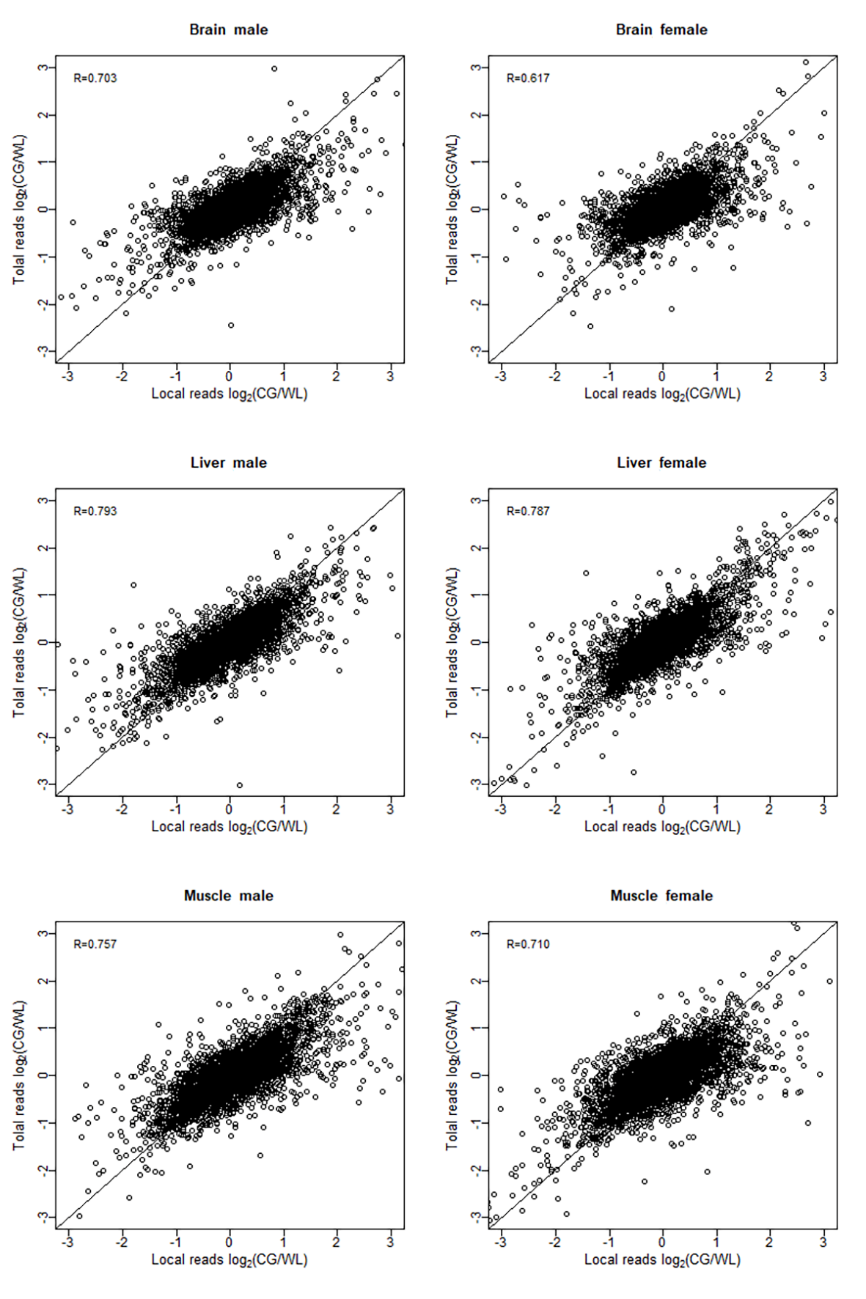


**Figure S2 Assessment of our local reads method for estimating allele specific expression.** Each point represents one gene. The x-axis shows fold change of local reads method, and the y-axis shows fold change of total reads method. Pearson correlation coefficient was calculated and tagged in the top left of each plot.


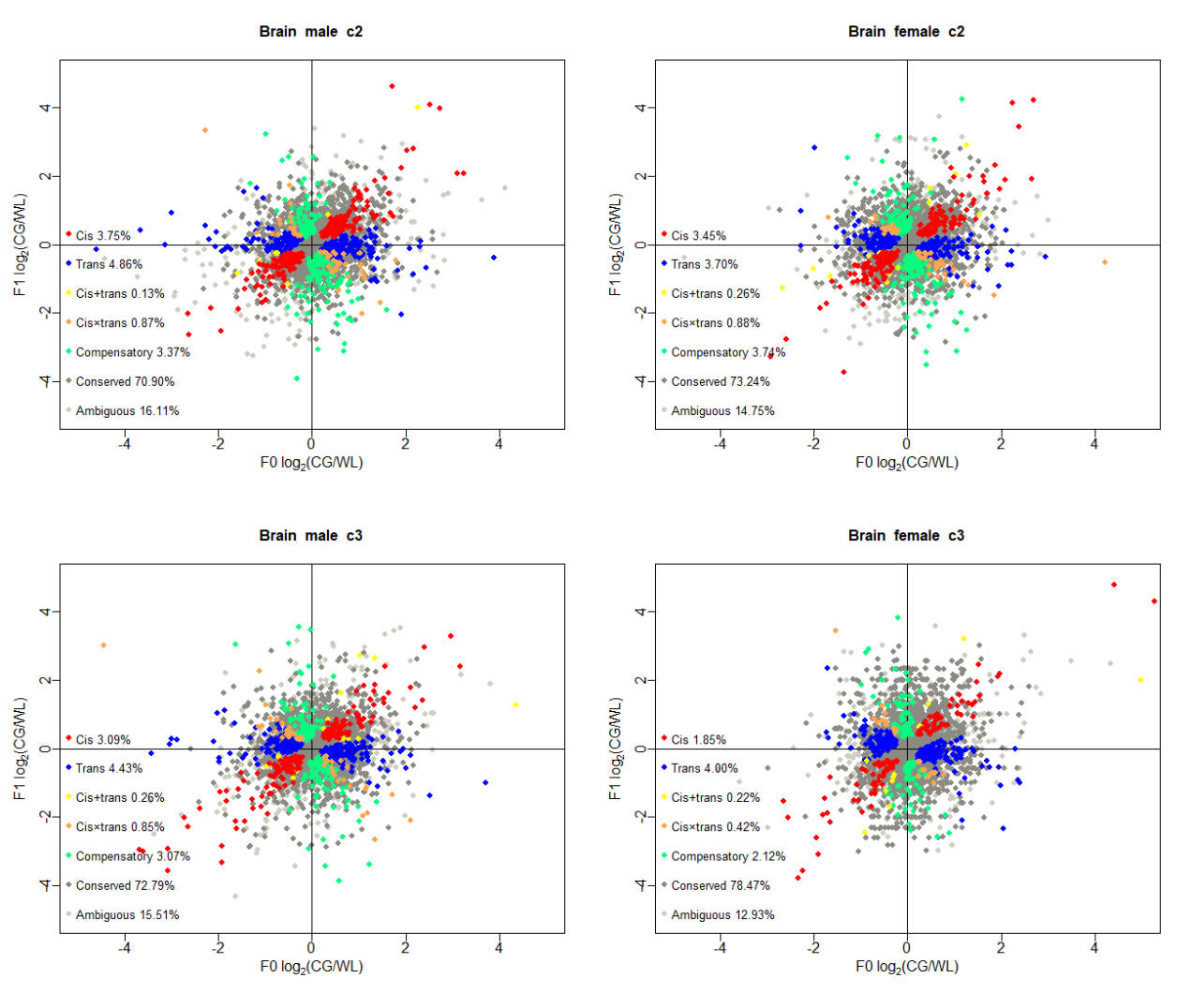


**Figure S3 Classification of genes in brain.** Each point represents a single gene and is color-coded according to its regulatory category. The coordinate position shows the average log2 expression fold change between the alleles in the hybrids (y-axis) and between the two purebreds (x-axis).


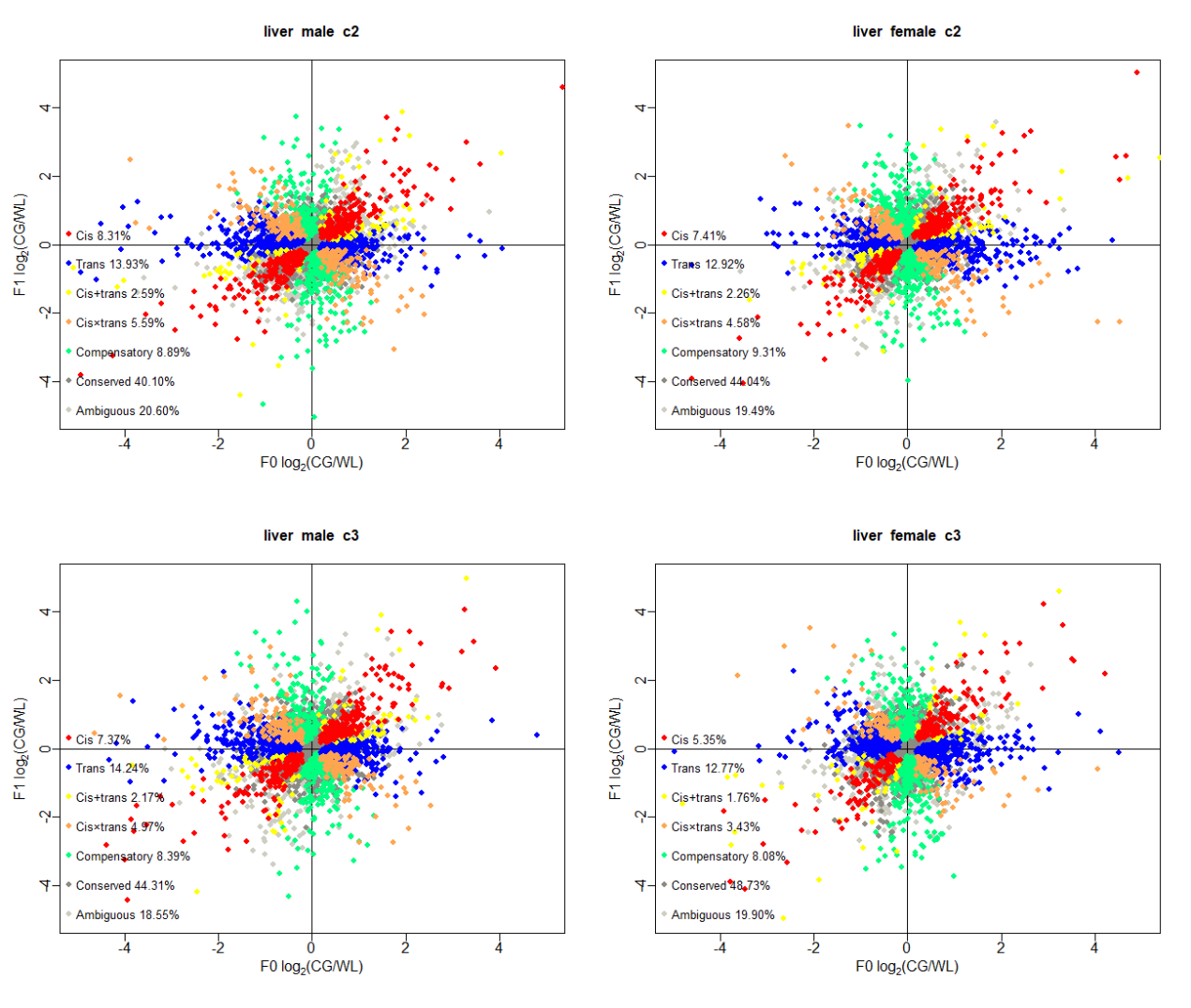


**Figure S4 Classification of genes in liver.** Each point represents a single gene and is color-coded according to its regulatory category. The coordinate position shows the average log2 expression fold change between the alleles in the hybrids (y-axis) and between the two purebreds (x-axis).


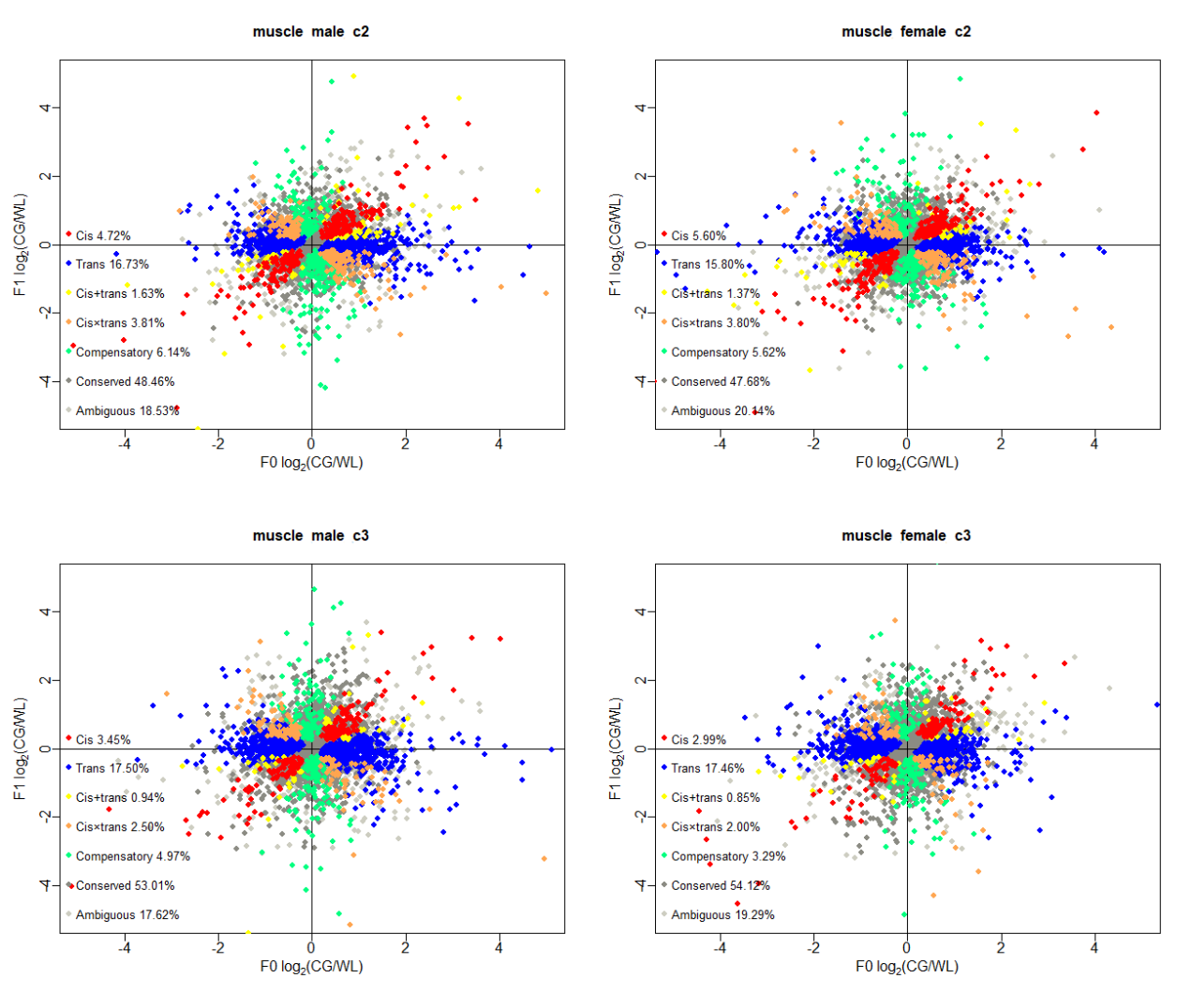


**Figure S5 Classification of genes in muscle.** Each point represents a single gene and is color-coded according to its regulatory category. The coordinate position shows the average log2 expression fold change between the alleles in the hybrids (y-axis) and between the two purebreds (x-axis).


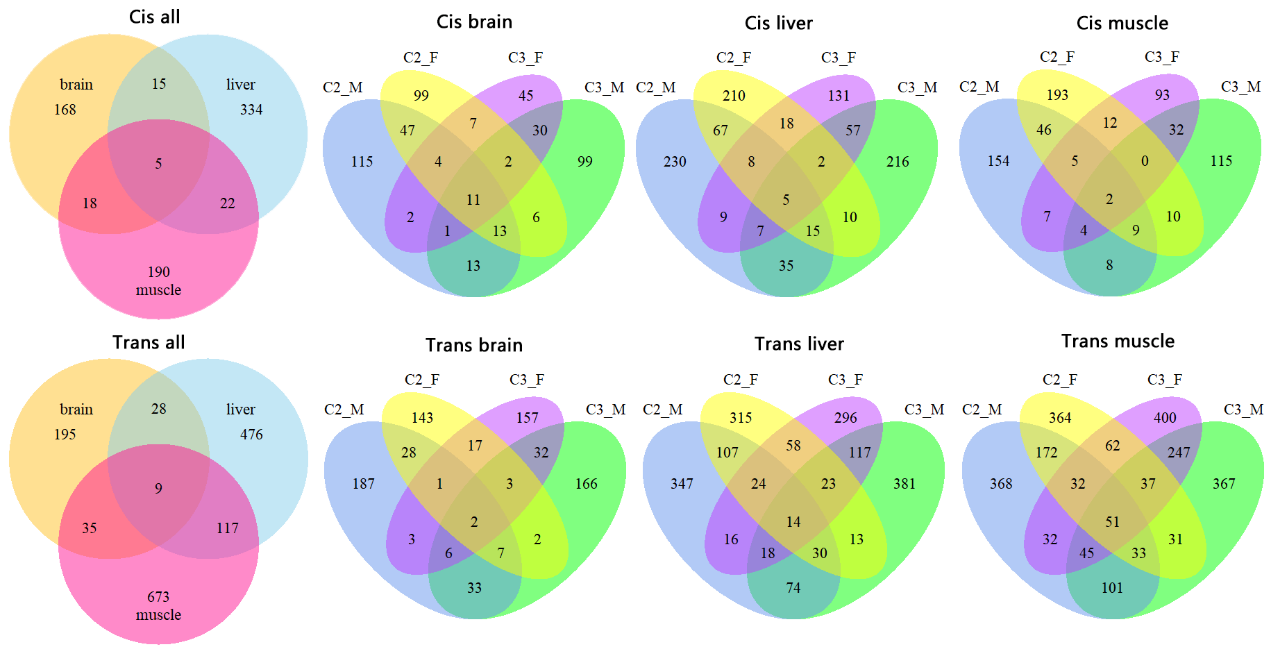


**Figure S6 Intersection of different groups of cis- and trans- regulatory genes.** For the two diagrams comparing different tissues (Cis-all, Trans-all), we only used the group of cross 2 males (C2_M). And for the rest diagrams, we compared cross 2 males (C2_M), cross 2 females (C2_F), cross 3 males (C3_M) and cross 3 females (C3_F) of each tissue.


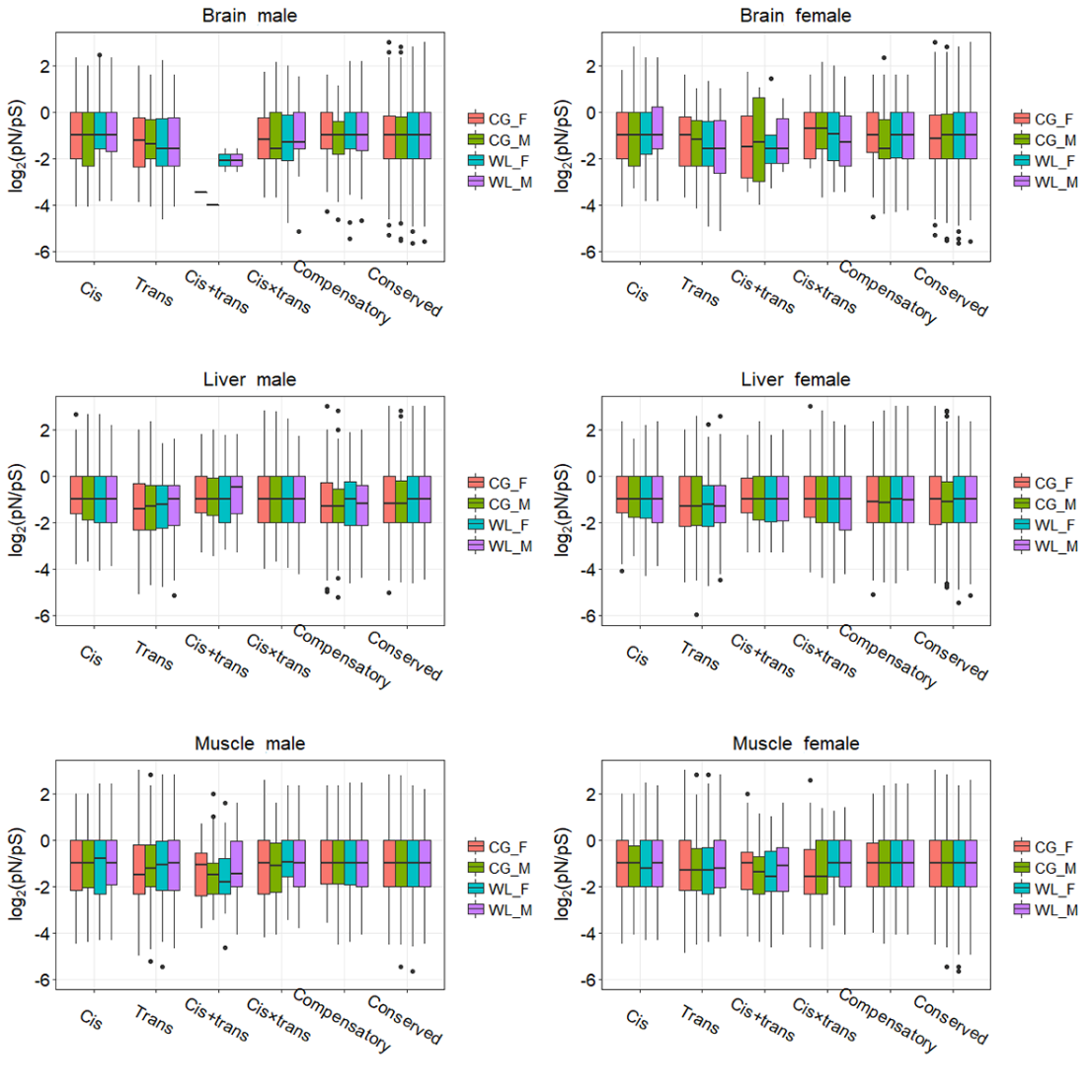


**Figure S7 The ratio of the numbers of non-synonymous SNPs to the numbers of synonymous SNPs (pN/pS) in different groups of cross 2.** The different colors represent the four parental genomes.


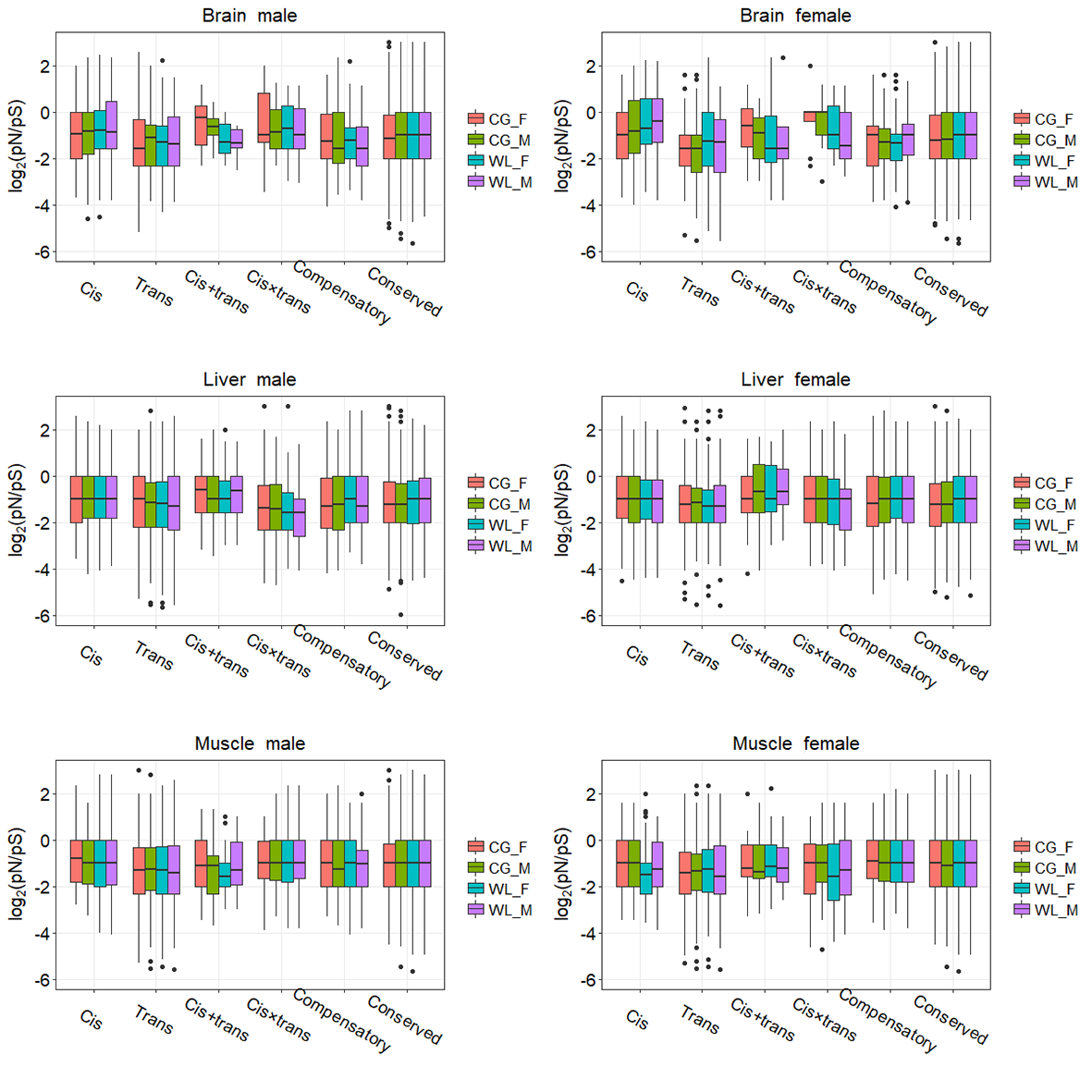


**Figure S8 The ratio of the numbers of non-synonymous SNPs to the numbers of synonymous SNPs (pN/pS) in different groups of cross 3.** The different colors represent the four parental genomes.

**Supplemental tables**

**Table S1 The summary of differential expression genes in hybrid and purebred progenies**

| Group | Total | Purebred | | |  | Hybrid | |
| --- | --- | --- | --- | --- | --- | --- | --- |
|  |  | Differential expression* | |  | Differential expression | | |
| b-2-m | 5492 | 969 | 17.64% |  | 801 | | 14.58% |
| b-2-f | 5485 | 807 | 14.71% |  | 856 | | 15.61% |
| b-3-m | 5666 | 952 | 16.80% |  | 754 | | 13.31% |
| b-3-f | 5528 | 854 | 15.45% |  | 319 | | 5.77% |
| l-2-m | 4524 | 1894 | 41.87% |  | 1477 | | 32.65% |
| l-2-f | 4521 | 1648 | 36.45% |  | 1426 | | 31.54% |
| l-3-m | 4706 | 1821 | 38.70% |  | 1399 | | 29.73% |
| l-3-f | 4433 | 1538 | 34.69% |  | 960 | | 21.66% |
| m-2-m | 4984 | 1886 | 37.84% |  | 1071 | | 21.49% |
| m-2-f | 4950 | 1900 | 38.38% |  | 1097 | | 22.16% |
| m-3-m | 5210 | 1787 | 34.30% |  | 743 | | 14.26% |
| m-3-f | 5190 | 1923 | 37.05% |  | 532 | | 10.25% |

*Differential expression was defined by binomial test, p-value <0.05. The purebred differential expression referred to the difference between two strains using the local reads covered the SNP list of cross 2 and cross 3, respectively. The hybrid differential expression referred to the difference between two alleles.

**Table S2 The difference of gene proportion of each categories between different groups**

| **p_value** | **b_2_m** | **b_2_f** | **b_3_m** | **b_3_f** | **l_2_m** | **l_2_f** | **l_3_m** | **l_3_f** | **m_2_m** | **m_2_f** | **m_3_m** |
| --- | --- | --- | --- | --- | --- | --- | --- | --- | --- | --- | --- |
| **b_2_f** | 0.91 |  |  |  |  |  |  |  |  |  |  |
| **b_3_m** | 0.99 | 0.93 |  |  |  |  |  |  |  |  |  |
| **b_3_f** | 0.33 | 0.19 | 0.71 |  |  |  |  |  |  |  |  |
| **l_2_m** | 0.14 | 0.15 | 0.29 | 0.04 |  |  |  |  |  |  |  |
| **l_2_f** | 0.22 | 0.29 | 0.46 | 0.088 | 0.99 |  |  |  |  |  |  |
| **l_3_m** | 0.21 | 0.18 | 0.45 | 0.12 | 0.99 | 0.99 |  |  |  |  |  |
| **l_3_f** | 0.15 | 0.16 | 0.47 | 0.31 | 0.85 | 0.95 | 0.96 |  |  |  |  |
| **m_2_m** | 0.018 | 0.0033 | 0.064 | 0.078 | 0.36 | 0.34 | 0.59 | 0.61 |  |  |  |
| **m_2_f** | 0.0556 | 0.0099 | 0.15 | 0.14 | 0.46 | 0.41 | 0.7 | 0.62 | 0.98 |  |  |
| **m_3_m** | 0.0024 | 0.00025 | 0.015 | 0.13 | 0.019 | 0.023 | 0.072 | 0.2 | 0.75 | 0.66 |  |
| **m_3_f** | 9.9E-05 | 2.76E-06 | 0.00055 | 0.0095 | 0.00087 | 0.00075 | 0.0044 | 0.011 | 0.34 | 0.3 | 0.91 |

The number in the table represents the p-value of Fisher’s exact test for the difference of gene proportion of each categories between different groups.

**Table S3 The gene list of intersection of each group**

| Group* | geneID | gene name |
| --- | --- | --- |
| c2_m_cis | ENSGALG00000004637 | SHPK, sedoheptulokinase |
|  | ENSGALG00000005004 | SAMD8, sterile alpha motif domain containing 8 |
|  | ENSGALG00000026253 | TMEM144, transmembrane protein 144 |
|  | ENSGALG00000032215 | ULK3, unc-51 like kinase 3 |
|  | ENSGALG00000042232 | PSMG4, proteasome assembly chaperone 4 |
| c2_m_trans | ENSGALG00000003523 | IFT52, intraflagellar transport 52 |
|  | ENSGALG00000011469 | IGFBP2, insulin like growth factor binding protein 2 |
|  | ENSGALG00000012072 | PARP14, poly(ADP-ribose) polymerase family member 14 |
|  | ENSGALG00000003999 | \ |
|  | ENSGALG00000004078 | LOC416263, LON peptidase N-terminal domain and RING finger protein 2-like |
|  | ENSGALG00000004140 | SH3BP4, SH3 domain binding protein 4 [ |
|  | ENSGALG00000011209 | EVL, Enah/Vasp-like |
|  | ENSGALG00000014570 | LPCAT3, lysophosphatidylcholine acyltransferase 3 |
|  | ENSGALG00000031895 | SDK2, sidekick cell adhesion molecule 2 |
| c2_f_cis | ENSGALG00000003991 | MRTO4, MRT4 homolog, ribosome maturation factor |
|  | ENSGALG00000013778 | EXOC1, exocyst complex component 1 |
|  | ENSGALG00000005004 | SAMD8, sterile alpha motif domain containing 8 |
|  | ENSGALG00000006141 | POFUT2, protein O-fucosyltransferase 2 |
|  | ENSGALG00000007519 | ACBD5, acyl-CoA binding domain containing 5 |
|  | ENSGALG00000013156 | ZNF830, zinc finger protein 830 |
|  | ENSGALG00000014435 | MRPL51, mitochondrial ribosomal protein L51 |
|  | ENSGALG00000030718 | \ |
|  | ENSGALG00000036257 | ORMDL1, ORMDL sphingolipid biosynthesis regulator 1 |
|  | ENSGALG00000039903 | VSIG10, V-set and immunoglobulin domain containing 10 |
| c2_f_trans | ENSGALG00000007810 | PPP4R2, protein phosphatase 4 regulatory subunit 2 |
|  | ENSGALG00000040340 | PCYOX1L, prenylcysteine oxidase 1 like |
|  | ENSGALG00000026607 | \ |
|  | ENSGALG00000030598 | BTAF1, B-TFIID TATA-box binding protein associated factor 1 |
|  | ENSGALG00000031214 | MED9, mediator complex subunit 9 |
|  | ENSGALG00000033385 | COPS8, COP9 signalosome subunit 8 |
| brain_cis | ENSGALG00000000399 | dual, specificity phosphatase 28 |
|  | ENSGALG00000006141 | POFUT2, protein O-fucosyltransferase 2 |
|  | ENSGALG00000043484 | LOC101747789, uncharacterized LOC101747789 |
|  | ENSGALG00000012400 | \ |
|  | ENSGALG00000029509 | NECAB1, N-terminal EF-hand calcium binding protein 1 |
|  | ENSGALG00000039903 | VSIG10, V-set and immunoglobulin domain containing 10 |
|  | ENSGALG00000042454 | DCDC2, doublecortin domain containing 2 |
|  | ENSGALG00000003549 | MTG1, mitochondrial ribosome associated GTPase 1 |
|  | ENSGALG00000004005 | \ |
|  | ENSGALG00000004637 | SHPK, sedoheptulokinase |
|  | ENSGALG00000005802 | FLT4, fms related tyrosine kinase 4 |
| brain_trans | ENSGALG00000001768 | TENM2, teneurin transmembrane protein 2 |
|  | ENSGALG00000014975 | DRD5, dopamine receptor D5 |
| liver_cis | ENSGALG00000001620 | CUTA, cutA divalent cation tolerance homolog |
|  | ENSGALG00000002277 | KLHL24, kelch like family member 24 |
|  | ENSGALG00000002699 | VAV2, vav guanine nucleotide exchange factor 2 |
|  | ENSGALG00000011900 | NAGA, alpha-N-acetylgalactosaminidase |
|  | ENSGALG00000029429 | TGFBI, transforming growth factor beta induced |
| liver_trans | ENSGALG00000000734 | RPA2, replication protein A2 |
|  | ENSGALG00000008828 | AFTPH, aftiphilin |
|  | ENSGALG00000030269 | CNIH4, cornichon family AMPA receptor auxiliary protein 4 |
|  | ENSGALG00000003923 | COL6A3, collagen type VI alpha 3 chain |
|  | ENSGALG00000004813 | IARS, isoleucyl-tRNA synthetase |
|  | ENSGALG00000006461 | UBE3C, ubiquitin protein ligase E3C |
|  | ENSGALG00000007434 | RHBDF1, rhomboid 5 homolog 1 |
|  | ENSGALG00000011114 | COG2, component of oligomeric golgi complex 2 |
|  | ENSGALG00000012207 | CCNT2, cyclin T2 |
|  | ENSGALG00000014570 | LPCAT3, lysophosphatidylcholine acyltransferase 3 |
|  | ENSGALG00000016834 | MCF2L, MCF.2 cell line derived transforming sequence like |
|  | ENSGALG00000034029 | \ |
|  | ENSGALG00000039606 | CLN8, ceroid-lipofuscinosis, neuronal 8 |
|  | ENSGALG00000042330 | PPP1R9B, protein phosphatase 1, regulatory (inhibitor) subunit 9B |
| muscle_cis | ENSGALG00000027096 | LGALSL, galectin like |
|  | ENSGALG00000033603 | METTL22, methyltransferase like 22 |
| muscle_trans | ENSGALG00000002524 | UCHL5, ubiquitin C-terminal hydrolase L5 |
|  | ENSGALG00000004618 | TAX1BP3, Tax1 binding protein 3 |
|  | ENSGALG00000009934 | NUP107, nucleoporin 107 |
|  | ENSGALG00000012159 | ACTR3, ARP3 actin related protein 3 homolog |
|  | ENSGALG00000013208 | CENPE, centromere protein E |
|  | ENSGALG00000016971 | TSC22D1, TSC22 domain family member 1 |
|  | ENSGALG00000032372 | CYB5R2, cytochrome b5 reductase 2 |
|  | ENSGALG00000035362 | \ |
|  | ENSGALG00000038325 | CORO7, coronin 7/PAM16 presequence translocase associated motor 16 homolog |
|  | ENSGALG00000041337 | OTUD6B, OTU domain containing 6B |
|  | ENSGALG00000045995 | \ |
|  | ENSGALG00000042773 | VOPP1, vesicular, overexpressed in cancer, prosurvival protein 1 |
|  | ENSGALG00000042895 | BCAR1, BCAR1, Cas family scaffolding protein |
|  | ENSGALG00000043240 | ARPP21, cAMP regulated phosphoprotein 21 |
|  | ENSGALG00000044661 | CTSG, cathepsin G |
|  | ENSGALG00000039193 | AGRN, agrin |
|  | ENSGALG00000039775 | SHC1, SHC adaptor protein 1 |
|  | ENSGALG00000040833 | CDCA7, cell division cycle associated 7 |
|  | ENSGALG00000040942 | EFCAB5, EF-hand calcium binding domain 5 |
|  | ENSGALG00000036017 | \ |
|  | ENSGALG00000036356 | YEATS2, YEATS domain containing 2 |
|  | ENSGALG00000037787 | MEF2D, myocyte enhancer factor 2D |
|  | ENSGALG00000038146 | TRIM58, tripartite motif containing 58 |
|  | ENSGALG00000033068 | TLR21, Toll-like receptor 21 |
|  | ENSGALG00000033513 | ELL, elongation factor for RNA polymerase II |
|  | ENSGALG00000033953 | SHROOM4, shroom family member 4 |
|  | ENSGALG00000034140 | ZNF395, zinc finger protein 395 |
|  | ENSGALG00000022891 | B4GALT3, beta-1,4-galactosyltransferase 3 |
|  | ENSGALG00000023682 | GTF2H3, general transcription factor IIH subunit 3 |
|  | ENSGALG00000026159 | PDLIM7, PDZ and LIM domain 7 |
|  | ENSGALG00000029678 | SNX16, sorting nexin 16 |
|  | ENSGALG00000013617 | PDGFRL, platelet derived growth factor receptor like |
|  | ENSGALG00000013872 | SEH1L, SEH1 like nucleoporin |
|  | ENSGALG00000014647 | RIT1, Ras like without CAAX 1 |
|  | ENSGALG00000016692 | CENPQ, centromere protein Q |
|  | ENSGALG00000012312 | GCAT, glycine C-acetyltransferase |
|  | ENSGALG00000012631 | PWP1, PWP1 homolog, endonuclein |
|  | ENSGALG00000012696 | \ |
|  | ENSGALG00000012849 | SLC37A3, solute carrier family 37 member 3 |
|  | ENSGALG00000011038 | CBX3, chromobox 3 |
|  | ENSGALG00000011621 | IGF2R, insulin like growth factor 2 receptor |
|  | ENSGALG00000011898 | FAM109B, family with sequence similarity 109 member B |
|  | ENSGALG00000012024 | MKL1, megakaryoblastic leukemia (translocation) 1 |
|  | ENSGALG00000006688 | TADA3, transcriptional adaptor 3 |
|  | ENSGALG00000007393 | SUDS3, SDS3 homolog, SIN3A corepressor complex component |
|  | ENSGALG00000007766 | KIAA1217, KIAA1217 |
|  | ENSGALG00000008551 | CRELD2, cysteine rich with EGF like domains 2 |
|  | ENSGALG00000003807 | GPN3, GPN-loop GTPase 3 |
|  | ENSGALG00000003939 | KLF2, Kruppel like factor 2 |
|  | ENSGALG00000004410 | ANAPC16, anaphase promoting complex subunit 16 |
|  | ENSGALG00000004583 | NPLOC4, NPL4 homolog, ubiquitin recognition factor |

*Each “group” in the table refers to the intersection of several groups, e.g., “c2_m_cis” represents the intersection of cis-regulated genes of group “brain_c2_m”, “liver_c2_m” and “muscle_c2_m”; “brain_cis” represents the intersection of cis-regulated genes of group “brain_c2_m”, “brain_c2_f”, “brain_c3_m” and “brain_c3_f”.
